# Gut-brain axis: Mechanisms and potential therapeutic strategies for ischemic stroke through immune functions

**DOI:** 10.3389/fnins.2023.1081347

**Published:** 2023-01-27

**Authors:** Sheng-Yu Zhou, Zhen-Ni Guo, Yi Yang, Yang Qu, Hang Jin

**Affiliations:** ^1^Department of Neurology, Stroke Center, The First Hospital of Jilin University, Changchun, China; ^2^Department of Neurology, Stroke Center & Clinical Trial and Research Center for Stroke, The First Hospital of Jilin University, Changchun, China

**Keywords:** ischemic stroke, gut-brain axis, gastrointestinal microbiome, immune system, therapeutic targets

## Abstract

After an ischemic stroke (IS) occurs, immune cells begin traveling to the brain and immune system from the gut and gastrointestinal tract, where most of them typically reside. Because the majority of the body’s macrophages and more than 70% of the total immune cell pool are typically found within the gut and gastrointestinal tract, inflammation and immune responses in the brain and immune organs require the mobilization of a large number of immune cells. The bidirectional communication pathway between the brain and gut is often referred to as the gut-brain axis. IS usually leads to intestinal motility disorders, dysbiosis of intestinal microbiota, and a leaky gut, which are often associated with poor prognosis in patients with IS. In recent years, several studies have suggested that intestinal inflammation and immune responses play key roles in the development of IS, and thus may become potential therapeutic targets that can drive new therapeutic strategies. However, research on gut inflammation and immune responses after stroke remains in its infancy. A better understanding of gut inflammation and immune responses after stroke may be important for developing effective therapies. This review discusses the immune-related mechanisms of the gut-brain axis after IS and compiles potential therapeutic targets to provide new ideas and strategies for the future effective treatment of IS.

## 1. Introduction

Ischemic stroke (IS) is a notable cause of morbidity and mortality worldwide and a leading cause of adult disability in developed countries (Johnson et al., 2016). Patients with IS often experience complications such as gastrointestinal bleeding, microbiota imbalance, and constipation, which accelerates disease progression and worsens prognosis (Benakis et al., 2016; Kapil et al., 2017; Chen et al., 2019c; Li et al., 2019; Fralick et al., 2020; Pluta et al., 2021). In recent years, researchers have amassed findings that strongly suggest a dynamic association between the gut-brain axis and IS; this topic may become a new research hotspot, which could expedite identification of potential therapeutic targets and eventually result in an intervention that could improve the prognosis of IS (Battaglini et al., 2020; Sinagra et al., 2021; Fang et al., 2022; Peh et al., 2022).

The gut possesses both digestive and immunomodulatory functions while also hosting a microbial ecosystem. The gut microbial ecosystem works with the host to regulate several seemingly unrelated processes: development and function of the nervous system, immune reactions, and metabolism. The gut and central nervous system (CNS) are intimately connected through the gut-brain axis, which allows for dynamic, bidirectional communication and interaction. The gut can regulate the structure and function of the brain, while the brain can, in turn, also affect the microenvironment and microbiota composition of the gut. As the gut contains over 70% of the inflammatory and immune cells in the body, an episode of IS can trigger mass migration of these cells to the brain, where they interfere with digestion and absorption of nutrients while simultaneously weakening the intestinal barrier. This often leads to poor outcomes in IS patients by affecting the development of their prognosis (Arya and Hu, 2018; Wiertsema et al., 2021).

The connection between the gut environment and the brain may also influence the host’s mood and behavior. Attempts to characterize the link between the gut microbiome and the brain are still underway; recent studies have begun to uncover some of the underlying mechanisms through which the brain affects gut barrier integrity and the gut microbiota influences the brain and subsequently the host’s behavior. Following cerebral infarction, studies have discovered not only changes in the gut microbiota but also changes in the manner in which this dysbiosis results in the systemic inflammation that eventually compromises CNS function (Chen et al., 2019b, 2021; Ghelani et al., 2021; Gwak and Chang, 2021). Post-IS outcome is believed to be affected by at least several factors, including the following: regulation of inflammation by CNS cells such as microglia, ingestion of oral probiotics, and fecal microbial transplantation (Holmes et al., 2020; Vendrik et al., 2020). However, due to the rapid and complex changes in immune status after IS, the current results of basic research and clinical trials targeting immunotherapy for IS are unsatisfactory, and several studies have pointed out that changes in the composition of the gut microbiota and the levels of its metabolites after IS affect the overall immune status of the host and are associated with IS prognosis. The results of basic research and clinical trials on immunotherapy for IS are promising (Sadler et al., 2020). Therefore, the modulation of post-IS immune status through the regulation of gut microbiota and its metabolite levels, as well as the modulation of related pathways and targets are expected to be new approaches for post-IS immunotherapy. This review focuses on the relationship between inflammation and the gut microbiota-brain axis, its relationship to IS, and therapeutic strategies that target the immune response of the gut-brain axis to provide new ideas and strategies for effective treatment of IS in the future.

## 2. Gut-brain axis

Between the brain and the gut, a bidirectional cross-communication occurs through multiple biological networks, including neural, immune, neuroendocrine, and metabolic pathways. Microbial changes in the gut are able to affect brain physiology and cognitive function through these pathways as well. Dynamic and bidirectional communication along the gut microbiota-brain axis can be mediated through several direct and indirect pathways, including the enteric nervous system and vagus nerve, neuroendocrine and hypothalamic-pituitary-adrenal (HPA) axis, the immune system, and microbiota-derived neuroactive compounds (Carabotti et al., 2015). As one of the significant component of the gut-brain axis, the gut microbiota can produce neurotransmitters such as dopamine, serotonin and γ-aminobutyric acid (GABA), which have regulatory effects on nerves and affect nerve function after IS; microbial metabolites such as short-chain fatty acids (SCFAs) and aryl hydrocarbon receptor (AhR) ligands, which changes after IS and leads to the stimulation of the innate immune system and the maturation of gut-associated lymphoid tissues; and amino acids (tyramine, tryptophan, etc.), which stimulate local and systemic immune responses, and plays an vital role in the clearance of drugs and toxins in the body (Carabotti et al., 2015; Zhu et al., 2017; Benakis et al., 2020; Li et al., 2020). The intestinal epithelium is composed of various specialized neuroendocrine cells that secrete hormones. These hormones and neuroactive compounds affect the local physiological functions of the gut, interact with the host immune system and metabolism, circulate through blood, and send direct signals to the brain. Gut microbiota can influence the integrity of the gut epithelial barrier, and control the signaling molecules transporting from the gut lumen to the lamina propria, which contains various immune cells and neurons or blood circulation. Some neuropsychiatric disorders disrupt the integrity of the gut barrier (Li et al., 2020). For example, stress can activate the HPA axis, resulting in the release of several hormones, including corticotropin-releasing hormone, adrenocorticotropic hormone, and cortisol. When IS occurs, cortisol modulates neuroimmune responses and affects gut barrier integrity. Thus, hormones, immune mediators, and CNS neurotransmitters produced during stress can alter the gut environment and microbiota composition.

### 2.1. Changes in gut microbiota composition and diversity after IS

Under physiological conditions, the intestinal microbiota of healthy adults is in dynamic balance almost all the time, with the most abundant microbiota being anaerobic bacteria and *Clostridium perfringens* (Tan et al., 2021; Chidambaram et al., 2022; Peh et al., 2022). In pathological conditions, however, the composition and diversity of the intestinal microbiota are altered. A study found that the abundance of intestinal *Streptococcus* spp. was significantly higher in unhealthy aging populations with previous major diseases than in healthy aging populations, and the composition of the intestinal microbiota was significantly altered and species diversity was significantly reduced in patients and experimental animals after the occurrence of IS (Singh et al., 2019). Haak et al. (2021) found that the intestinal microbiota alpha diversity and beta diversity were reduced and the abundance of *Aspergillus*, Enterobacteriaceae, and *Escherichia coli* spp. were significantly higher in patients with IS compared to controls. In contrast, in the animal model of IS, the trends of intestinal microbiota diversity, *Aspergillus* and Enterobacteriaceae were similar to those of IS patients (Ahnstedt et al., 2020; Blasco et al., 2020; Xu et al., 2021). Xu et al. (2021) illustrated that the composition and diversity of the intestinal microbiota changed significantly after IS, and that the most prominent change in the intestinal microbiota after IS was in Enterobacteriaceae, whose abundance was significantly higher in the acute phase of IS. The overgrowth of Enterobacteriaceae can exacerbate brain infarction by increasing systemic inflammation and is an independent risk factor for primary adverse outcomes of patients with stroke. In both humans and experimental animals, IS reduced intestinal microbiota diversity and increased the abundance of the Enterobacteriaceae (Ahnstedt et al., 2020; Blasco et al., 2020). The reason may be that the acute stress of IS induces intestinal ischemia, and the ischemic intestine produces large amounts of nitrates in response to free radicals, thus favoring the growth of the nitrate-responsive microbiota of the Enterobacteriaceae, which in turn aggravates the inflammatory response and is detrimental to the prognosis of IS. In a study with the pig IS model, Jeon et al. (2020) collected fecal samples pre-stroke and 1, 3, and 5 days post-stroke to assess changes in the gut microbiome. The results showed that the most significant changes in microbial patterns were observed between pre-stroke and 3 days post-stroke. Compared with pre-stroke, the abundance of Proteobacteria increased significantly 3 days post-stroke, while that of Firmicutes decreased. The abundance of the lactic acid bacteria *Lactobacillus* decreased 3 days after stroke. By day 5, the microbial pattern returned to values similar to those before stroke. This finding provides a basis for describing gut microbial changes in the acute phase of stroke and can be used to assess the potential evolution of stroke pathology and therapeutic targets.

The gut microbiota may also play a vital role in post-IS complications such as infection, cognitive impairment, depression, muscle wasting and weight loss. a study by Stanley et al. (2016), which collected adequate clinical and preclinical evidence, proposed that there is translocation and transmission of commensal bacteria in the host intestinal microbiota after the onset of IS. In a mouse model of IS combined with diabetes constructed by Sun et al. (2016a), probiotic supplementation attenuated ischemic brain injury and cognitive dysfunction in mice. Since changes in the composition of intestinal microbiota can coincide with changes in body weight, cachexia, protein catabolism in skeletal muscle, and mood dysregulation in other conditions, it is hypothesized that intestinal microbiota may also be associated with depression and muscle atrophy after IS. Therefore, intervention in the composition and diversity of the gut microbiota may be considered as a therapeutic approach for the treatment of IS.

### 2.2. Changes in gut microbiota metabolites after IS

The levels of gut microbiota metabolites including trimethylamine oxide (TMAO), SCFAs and other substances are also affected by IS. Dietary nutrients such as choline can be specifically produced by intestinal microbiota as trimethylamine (TMA), which is absorbed by the intestine and converted to TMAO by hepatic monooxygenase. after IS, plasma TMAO levels in patients show a dynamic change with an increase in the acute phase followed by a decrease and a continuous increase in the chronic phase (Schneider et al., 2020). It was found that plasma TMAO levels were significantly higher in patients with moderate to severe IS [defined by National Institute of Health Stroke Scale (NIHSS) scores equal to 6] than in patients with mild IS, and that elevated plasma TMAO levels in the acute phase were associated with increased infarct volume (Wu C. et al., 2020). High plasma TMAO levels not only correlate with infarct size and severity of IS, but also increase the risk of IS recurrence (Haghikia et al., 2018). Increased abundance of TMA-producing bacterial spp. in the intestinal microbiota of patients with IS may explain the high plasma TMAO levels, while decreased abundance of butyric acid-producing bacteria may also suggest changes in the levels of SCFAs after IS (Ahnstedt et al., 2020; Haak et al., 2021). Short-chain fatty acids are produced by intestinal microbiota metabolizing indigestible fibers, including acetate, butyric acid, and propionic acid, and can exert intestinal stabilizing effects through various pathways and have recently been shown to cross the blood-brain barrier (BBB) (Smith et al., 2013; Lee et al., 2020). Fecal SCFAs levels were found to be significantly reduced after the onset of IS in patients and animal models of IS, and plasma SCFAs levels were also significantly reduced in animal models, and fecal SCFAs levels were negatively correlated with neurological function scores and brain infarct volume after the onset of IS in both population studies and animal experiments (Chen et al., 2019c; Tan et al., 2021). The above results suggest that direct regulation of microbiota metabolite levels such as TMAO and SCFAs may become one of the methods to improve the prognosis of IS.

## 3. Involvement of the gut-brain axis in post-IS inflammation and immune responses

Recent evidence suggests that intestinal inflammation and immune responses after IS are promising therapeutic targets due to their critical roles in the progress of stroke pathophysiology. After IS, ischemic brain tissue generates injury-related molecular patterns that initiate local and systemic innate and adaptive immune responses through pattern recognition receptors, such as Toll-like receptors (TLRs). Innate immune cells, such as neutrophils, macrophages and their CNS counterparts, microglia, mast cells, innate lymphocytes [e.g., interleukin (IL) -17-secreting γδ Tregs (T cells)], natural killer Tregs, respond within hours and subsequently activate T and B lymphocytes (Lee and Kim, 2017). T-cell subsets can aggravate or attenuate the injury in ischemic brain tissue. For example, T helper cell (Th) 1, Th17, and γδ Tregs are pro-inflammatory immune cells, which is commonly associated with promoting inflammatory injury, while regulatory Tregs increase the secretion of IL-10, an anti-inflammatory cytokine, to suppress inflammation after IS (Luckheeram et al., 2012; Mittrücker et al., 2014; Zhu, 2018; Ruterbusch et al., 2020).

Gut microbiota is required for normal development of both the intestinal and peripheral immune systems as well as the brain. For example, gut microbiota affects microglia. Germ-free mice have a majority of immature microglia, and administration of a complex microbiota or SCFAs can promote microglial maturation and correct mature microglial deficiency (Erny et al., 2015). Microglia influenced by gut microbiota influence human behavior and neurological disease processes. Cytokines produced in the gut can circulate through the bloodstream into the brain, affecting the local and systemic immune system. Altered immune signaling in the brain, these cytokines has been proved that are linked to depression, anxiety, autism and other neuropsychiatric disorders (Emsley et al., 2008; Wang et al., 2014; Franke et al., 2021; Korostynski et al., 2021; Roth et al., 2021).

The metabolites and structural components of intestinal microbiota can affect the brain-gut axis, thereby promoting or inhibiting the process of inflammation and brain injury after IS. Lipopolysaccharide (LPS) is a major component of some microbial cell walls. LPS produced by gut microbes promotes the expression of proinflammatory cytokines and increases the permeability of the BBB, which is impaired and ultimately leads to neurological damage after IS (Zheng et al., 2019; Liao et al., 2020; He et al., 2021; Li F. et al., 2021). Thus, gut microbiota joins injury, infection, and autoimmunity as one of the few with the capability to increase permeability of the BBB, thereby increasing passage of circulatory microbial products into the brain and triggering a systemic inflammatory response. Immunosensitization of the BBB is a consequence of neuropathological symptoms. that may suggest a link between the gut environment and the brain, supporting theories of the gut-brain axis. In addition, inflammatory factors such as IL-1 and IL-6 that are expressed in the hypothalamus can induce the release of cortisol by activating the HPA axis, which links neuroimmunity with neuroendocrine activity. Increasing strain of post-IS through stress responses (Kozak et al., 1998; Smith et al., 1999; Dunn, 2000). This may affect the pathological process of IS, providing new therapeutic ideas for intervention in the neuroinflammatory response after IS.

### 3.1. Gut microbiota regulates neuroinflammation and immune responses

When IS occurs, damage-associated molecular patterns (DAMPs) and cytokines produced by cerebral ischemic tissue are released into circulation and enter immune organs, leading to systemic inflammation and immune responses (Dabrowska et al., 2019; Kumar, 2019; Jiang et al., 2021; Klegeris, 2021; Stanzione et al., 2022). For example, high mobility group protein B1 (HMGB-1) is a member of DAMP molecule. HMGB-1 produced in stroke-injured tissue can enter circulation through the BBB with increased permeability, thereby initiating post-stroke systemic inflammatory response syndrome (Roussel et al., 2011). Among them, tumor necrosis factor-α (TNF-α), interferon-γ (IFN-γ), and IL-6 are increased after stroke, and this process may be explained by “cytokine storm” (Tao et al., 2015; Xia et al., 2018; Zhu et al., 2018; Vogelgesang et al., 2019). Inflammation and immune responses after IS can be mediated by DAMPs alone in the absence of microbial infection and by both DAMPs and pathogen-associated molecular patterns (PAMPs) when IS is complicated by microbial infection (e.g., gut-derived sepsis) (Pirozhkov et al., 2018; Colonna, 2019; Clark and Vissel, 2021; Fischer et al., 2021). In general, the innate immune response emerges first by innate immune cells, including neutrophils, microglia, and macrophages, mast cells, IL17-secreting γδ Tregs, and NK cells. The systemic inflammatory response and immune response subsequently trigger the activation of adaptive immune cells after IS, while the adaptive immune response is mainly mediated by T and B lymphocytes (Gelderblom et al., 2012, 2018).

#### 3.1.1. Gut microbiota affects innate immunity after IS

Activation of intrinsic immunity can significantly affect the development and prognosis of IS, and intestinal microbiota can also influence the process of intrinsic immunity after IS. During the hyperacute phase of IS, intracytoplasmic calcium overload in microglia of brain parenchyma triggers the release of proinflammatory factors such as IL-1β and IL-18, which aggravate ischemic brain injury (Poh et al., 2019). 24 h after IS, a large number of monocytes associated with cerebrovascular infiltration of brain parenchyma can be seen and peak at day 3 (Su et al., 2017). The genes related to signal transduction pathways such as TREM1 and JAK/STAT were upregulated in post-IS infiltrated brain tissue compared with circulating blood macrophages, which phagocytosed and cleared dead neurons together with microglia during the subacute phase of IS (Zhang W. et al., 2019). The phagocytosis of neutrophils in infiltrating brain ischemic tissues and blood vessels in the injury area is decreased, generating high levels of reactive oxygen species and aggravating brain injury. In contrast, the intrinsic immune cells that infiltrate brain tissue after the onset of IS can be derived from the intestine (Ritzel et al., 2018; Otxoa-de-Amezaga et al., 2019). A study by Brea et al. (2021) found the temporal and spatial differences in the transfer of intestinal immune cells to the periphery using photoconversion techniques. At day 3 of IS, intestine-derived CD11c^+^ cells tended to transfer to brain tissue and meninges more than to immune organs, and the migration of intestine-derived CD11c^+^ cells showed tissue specificity, as demonstrated by the fact that CD11c^+^ cells transferred from intestine to meninges were mainly macrophages, while CD11c^+^ cells transferred from intestine to brain tissue contained macrophages and dendritic cells. On day 14 of IS, after the acute inflammatory response subsided, spatial variability in the transfer of intestinal immune cells to the periphery disappeared.

Injured blood vessels after stroke can stimulate the expression of cell surface molecules, including chemokines, adhesion molecules, intercellular adhesion molecule 1, and vascular cell adhesion molecule 1, to promote leukocyte adhesion and passage across the BBB (Zee et al., 2007; Sardari et al., 2021; Yu et al., 2022). After IS occurs, microglia are activated, cytokines such as IL-4, TNF-α, and IL-1β are released by damaged neurons and activated microglia; and the site of infarction is infiltrated by immune cells including neutrophils, macrophages, lymphocytes, and DCs (Chen S. et al., 2017; Chen et al., 2019a; Hsu et al., 2020; Liu et al., 2020). Microglial activation is the earliest stage of cellular inflammatory change after IS in this process. Several studies have shown that in animal models of middle cerebral artery occlusion (MCAO), microglia peak at 48 h and remain stable at 96 h after MCAO, whereas neutrophils migrate into the cortical brain parenchyma at 24 h post-IS (Ye et al., 2019; Ran et al., 2021); infiltration of neutrophils into the injury site can also be seen as an early event after IS (Ritzel et al., 2015; Zhang B. et al., 2019).

Immune status after IS can be influenced by modulating the level of intestinal microbiota and its metabolites. Acquired intestinal microbiota colonized germ-free mice showed increased numbers and massive activation of microglia and macrophages in the ischemic hemisphere and upregulation of transcription of pro-inflammatory factors such as IL-1β and tumor necrosis factor-α compared to germ-free mice (Singh et al., 2018). The increase in plasma TMAO level by dietary intervention increased the level of pro-inflammatory monocytes in the blood of mice, but the effect was lost after the removal of intestinal microbiota (Battaglini et al., 2020). However, supplementation with SCFAs significantly reduced the total number of microglia in the mouse brain and resulted in a hypoactivated morphology with increased branching and reduced sphericity (Sadler et al., 2020). These results suggest the need for selective modulation of intestinal microbiota and metabolite levels.

#### 3.1.2. Gut microbiota affects adaptive immunity after IS

After IS occurs, intestinal microbiota dysregulated, and T cell subsets (Th1, Th2, Th17, and Treg) are unbalanced, triggering various autoimmune and inflammatory responses. Among these cells, Th1 cells, as pro-inflammatory cells, secrete IL-2, IL-12, TNF-α, IFN-γ, and other pro-inflammatory cytokines to promote cellular immune response, which may be involved in the pathogenesis of nerve injury and inflammation after IS. Th2 cells secrete IL-4, IL-5, and IL-13 to promote humoral immune responses against parasites and allergens, which is generally not involved in the progress of pathophysiology after IS. IL-17 is produced by IL-17-secreting αβTregs (Th17 cells), which are required for antigen-specific priming. It has been previously theorized that IL-17 is mainly produced by Th17 cells during acute infection and does not require prior antigen priming. Therefore, IL-17 can rapidly induce inflammation in the body, resulting in more severe injury and inflammation (Benakis et al., 2016). In experimental cerebral ischemia, IL-17 production by γδ Tregs exacerbates the pro-inflammatory response (Gelderblom et al., 2012, 2018; Waisman et al., 2015). Benakis et al. (2016) showed that γδ Tregs are abundant in the gut, and only transported to the pial structures of the brain after stroke. Derived from same lineage as naive CD4 Treg, Treg cells can express Foxp3, a transcription factor, and secrete IL-10 to suppress excessive immune responses. A study by Liesz et al. (2009), using an animal model for IS, showed that loss of Treg cells enhanced post-stroke activation of resident and invading inflammatory cells, including microglia and Tregs. Treg cells also inhibit differentiation of Th17 cells and proliferation of γδ Tregs in the gut in order to exert anti-inflammatory effects. Treg cells play a key role in suppressing IS (Liesz et al., 2009; Na et al., 2015).

After IS, Tregs infiltrate the ischemic cerebral hemisphere. Tregs infiltrate the site of infarction from day 1, gradually increase in number from day 7, and peaked on day 14 (Grønberg et al., 2013). Xie et al. (2020) found that in a mouse model of IS, CD4 + and CD8 + T cells in the peri-infarct area increased for up to 1 month, whereas in patients with IS, the increase in Tregs persisted for several years. Studies have shown that CD4 + CD25 + Foxp3 + Treg cells, which play an important regulatory role in the anti-inflammatory process, can accumulate in ischemic lesions for 14–30 days, exert anti-inflammatory effects after the occurrence of IS, and protect brain tissue by suppressing effector Tregs. In the absence of Treg cells, in the presence of activation, the number of inflammatory cells such as microglia and Tregs that reside and infiltrate the infarcted area increases. Selvaraj et al. (2021) found a significant increase in the number of CD4^+^ T and CD8^+^ T cells in the ischemic hemisphere in mice at days 14 and 30, which was associated with a worse functional prognosis. However, anti-CD4 + T-cell treatment had no significant effect on infarct formation and mortality in mice, while cutting the number of CD8 + T cells in the chronic phase of IS improved the functional prognosis of IS. the level of peripheral adaptive immune cells changes dynamically after the onset of IS, and changes in Tregs correlate with IS severity and prognosis (Weitbrecht et al., 2021). The study of Li S. et al. (2021) found that blood B cells as a percentage of lymphocytes, CD8^+^ T cells as a percentage of lymphocytes, and CD8 + T cells as a percentage of lymphocytes in patients with IS and CD8 + T cells as a percentage of lymphocytes both showed elevated and then maintained high levels, while CD4^+^ T cell percentage showed a dynamic change of elevation and then recovery. In particular, the percentage of CD4^+^ Tregs showed a dynamic change of decrease, increase, recovery, and increase again at admission, discharge, 1 month of onset, and 3 months of onset. Among these, reduced CD4^+^ Tregs levels at admission were associated with worse neurological function at admission, worse NIHSS scores and mRS scores at discharge in IS patients, and were good predictors of mRS scores at 3 months. Xia et al. (2021) demonstrated that expansion of CD4^+^ Tregs cell numbers after specific intervention in mice reduced brain infarct volume and neuronal death, and facilitated long-term prognosis. This result shows the importance of CD4^+^ Tregs cells, while the role of B cells on IS still needs to be further investigated.

Researches on animal models suggest that increased numbers of infiltrating CD4^+^ T cells in brain tissue after IS are associated with poor prognosis of stroke, but anti-CD4^+^ T cells do not improve the severity of infarction (Zhang et al., 2022). In contrast, results from population-based cohorts suggest that decreased numbers of CD4^+^Tregs in the peripheral circulation are associated with poor prognosis in IS (Dolati et al., 2018). The conflicting results of several studies suggest the complexity of CD4^+^T cells in the IS process, with another subpopulation of CD4^+^T cells, Th17 cells, also gaining attention. After IS, although the number of CD4^+^T cells increases in both brain tissue and circulating blood, the Tregs/Th17 ratio decreases, accompanied by increased levels of the pro-inflammatory factor IL-17 and decreased levels of the anti-inflammatory factor transforming growth factor-β. In contrast, restoration of Tregs/Th17 ratios in brain tissue and circulating blood correlates with reduced infarct size and long-term functional prognosis after IS (Dolati et al., 2018; Zhang et al., 2022). Therefore, the role played by CD4^+^ T cells, Tregs cells, and Th17 cells on the prognosis of IS after IS needs to be considered in a comprehensive manner and further studies are still needed.

### 3.2. Specific immune-related pathways in the post-IS gut-brain axis

#### 3.2.1. LPS/TLR4

Cellular components derived from the gut microbiota influence CNS and enteric nervous system (ENS) function by activating TLRs expressed in gut neurons, sensory afferent neurons, and other cells of the brain (Brea et al., 2011; Burgueño et al., 2016; Leitner et al., 2019; Tajalli-Nezhad et al., 2019). LPS is a component of Gram-negative bacterial cell wall and is a substance composed of lipids and polysaccharides. It is an endotoxin that induces inflammation by activating TLRs with the help of LPS-binding protein (LBP) and CD14. In animal experiments by Kawato et al. (2013) it was mentioned that continuous intravenous administration of Gram-negative bacteria may promote stroke in rats prone to spontaneous hypertensive stroke, mainly caused by LPS-induced oxidative stress. In brain tissue, microglia and astrocytes are the main cell types capable of expressing TLRs, especially TLR4. Thus, LPS released by gut microbes after IS induces TLR4 expression in both microglia and astrocytes, and triggers the inflammatory response after IS. LPS/TLR4 signaling in microglia increases the expression of inflammatory cytokines in the CNS and gut and also affects neuronal survival in the ENS (Abg Abd Wahab et al., 2019; Kurita et al., 2020; Yang et al., 2021). Compared to microglia from conventional mice, immature microglia were found to be less responsive to LPS in germ-free mice. Lipid A and core LPS signals have been observed at the blood-brain interface, and co-localization of LPS with CD14/TLR4 receptors and lipoprotein receptors [including SR-BI and low-density lipoprotein (LDL) receptor], suggesting that LPS enters the brain under physiological conditions, possibly through a lipoprotein transport mechanism (Janova et al., 2016; Leláková et al., 2020). A study in which an LPS injection was administered to pigs supported the general idea that LPS affects cytokine and neurotransmitter levels; the LPS injection was found to increase expression of several pro-inflammatory cytokines (IL-1 receptor agonists, IL-6, TNF-α, and IFN-γ) and decrease norepinephrine levels in the cortex of the hypothalamus, hippocampus, and frontal lobe (Dickinson et al., 2009)Another study reported that compared with control mice, LPS exposure increased the expression of TNF-α, COX-2, NOS-2 and IL-1β, activated TLR4 and NF-κB signaling, and induced neuroinflammation in the cortex and hippocampus of adult mice (Luo et al., 2021; Xian et al., 2021). Other studies have confirmed that LPS can induce neuronal degeneration by increasing Bax/Bcl2 levels, decreasing cytochrome-c, and decreasing the expression of poly ADP-ribose polymerase-1 (Iványi et al., 2003). However, TLR4 is also important for the activation of vascular endothelial cells and microglia after IS and for neuroprotection against experimental brain injuries. They also reported reduced neuronal cell mortality and reduced lesion volume in LPS-injected mice after experimental brain injury, suggesting that peripheral LPS injection may reduce nerve damage by inducing a transient inflammatory response and providing neuroprotection without causing obvious adverse effects. Activation of TLR4/P13K/Akt/MAPKs pathway induces matrix metalloproteinase 9 (MMP-9) expression in astrocytes and astrocytes migration, leading to intestinal leakage and increased BBB permeability (Xian et al., 2021; Yang et al., 2021). LPS induces neuroinflammation and cognitive impairment in mice by activating microglia and NF-κB pathways as well as inducing amyloid production, memory dysfunction, and neuronal cell death. In addition, LPS appeared to increase levels of nitric oxide, prostaglandin E2, IL-1β, TNF-α, and Aβ1-42, while decreasing the levels of IL-10 and IL-4 (Xian et al., 2021). These findings demonstrated that after migrating from the intestine to the brain, LPS can induce neuroinflammation through circulation, which in turn triggers post-IS neuroinjury. It is noteworthy that LPS has both beneficial and detrimental effects on intestinal and central neurons, depending on its concentration. LPS can be harmful and trigger inflammation in the brain due to the presence of TLR4 polymorphisms, or when the LPS concentration is higher than the physiological threshold, TLR4 is more sensitive to physiological concentrations of LPS (Kayagaki et al., 2013; Ciesielska et al., 2021; Sangaran et al., 2021). Interestingly, several studies have shown that low-dose of LPS preconditioning can confer a protective state of apoptosis upon subsequent stimulation with high LPS concentrations, suggesting that TLR4 preactivation may play a role in the LPS-induced neuroprotective signaling pathway (Vartanian et al., 2011; Sangaran et al., 2021).

#### 3.2.2. SCFA receptor and AhR signaling pathway

Short-chain fatty acids are major metabolites produced by bacterial fermentation of dietary fibers in the gastrointestinal tract. They can regulate immune homeostasis in the gut, peripheral immune system, and brain (Desbonnet et al., 2010; Dalile et al., 2019; Silva et al., 2020). SCFAs receptors have been shown to exist on the surface of microglia, suggesting that SCFAs can affect microglial function (Erny et al., 2015). After IS, SCFAs in ischemic tissue can promote the phenotypic transition of microglia and promote neurogenesis and neurite outgrowth licensing effects by activating BDNF-TrkB signaling (Hasan et al., 2013; Jaworska et al., 2019). SCFAs also promotes the expression of anti-inflammatory genes and the downstream IL-10/STAT3 pathway in microglia by inhibiting histone deacetylase, thereby promoting nerve regeneration and axonal growth, promoting microglia through the GSK3β/PTEN/Akt axis, polarizing macrophages to the protective type, and protecting the white matter after IS (Patnala et al., 2017). In addition, SCFAs, metabolites of tryptophan, control astrocyte and microglia activation and modulate neuroinflammation through AhR signaling (Rothhammer et al., 2018). AhR protein levels are upregulated after IS mediates acute nerve injury at ischemic sites and activates pro-inflammatory gliosis, both of which slow down neuronal regeneration in subacute or chronic phases (Chen et al., 2019d). In DCs, AhR inhibits proinflammatory cytokines and promotes transforming growth factor (TGF)-β-driven Treg cell production. In Tregs, AhR induces Th17 differentiation and stabilizes Tregs and Tr1 cells. In intraepithelial lymphocytes, AhR downregulates the T co-inducing factor POZ/Krüppel-like factor to differentiate into immunomodulatory TCRαβ^+^CD8αα^+^ intraepithelial lymphocytes. AhR activation in myelin-specific CD2^+^CD5^+^ intraepithelial lymphocytes induce migration to the CNS and limits inflammation via the lymphocyte-activating gene 3 protein and TGF-β (Benson and Shepherd, 2011). AhR mediates ILC3 homeostasis and IL-22 secretion, and AhR deficiency reduces ILC3 levels and protects against *Citrobacter rodentium* infection. Thus, intestinal AhRs can control inflammation in distant CNS organs as well as in the gut. In astrocytes and microglia in the CNS, AhR activation inhibits a pro-inflammatory program driven by NF-κB. These findings suggest that the function and phenotype of immune cells in the brain can be influenced by gut microbiota metabolites, further affecting post-stroke neuroregeneration.

#### 3.2.3. TMAO/NLRP3

Trimethylamine lyase is widely expressed in intestinal microbiota and metabolizes dietary nutrients in eggs, liver, beans, and other foods to form TMA. TMAO is the oxidation product of TMA, which is absorbed by the passive diffusion of TMA through the cell membrane and enters the liver through the liver-intestinal circulation, where it is produced under the catalysis of hepatic flavin monooxygenase (FMO) (Zhang et al., 2020b). Higher levels of TMAO in plasma and cerebrospinal fluid have been shown in several studies to contribute to the development of stroke risk factors and induce cerebrovascular disease, and TMAO can be regarded as an important indicator of myocardial infarction, atherosclerosis, and stroke (Zhao and Wang, 2020; Praveenraj et al., 2022). Different SCFAs products, lactic acid and α-ketoglutarate play important roles in the synthesis of neurotransmitters such as glutamate, GABA, 5-HT and catecholamines. Increasing their concentration may lead to increased TMA accumulation and TMAO synthesis. Tmao-mediated pathogenesis involves activation of inflammatory signaling pathways, such as nuclear factor kappa B (NF-κB), pyrin dome-containing protein 3 (NLRP3) inflammasome, and the MAPK/JNK pathway in the peripheral and central nervous systems. Studies have shown that TMAO levels with increasing age induce mitochondrial dysfunction, oxidative stress and brain synaptic damage, causing endothelial dysfunction, BBB permeability and neuronal degeneration, leading to increasing TMAO-related cognitive impairment (Chen M. L. et al., 2017; Wu K. et al., 2020; Zhang et al., 2020b; Zhao and Wang, 2020; Praveenraj et al., 2022). Further studies are needed on the relationship between dietary food consumption and gut microbiota-dependent TMAO levels may provide new therapeutic options and strategies for IS and other neurological disorders.

#### 3.2.4. HPA axis pathway

The HPA axis constitutes a major part of the neuroendocrine system and regulates body functions in response to stress stimuli mainly through the vagus nerve in the brain-gut axis. The quality and quantity of gut microbiota and the expression of TLRs may influence neuroendocrine secretion and are critical for the HPA axis in stress responses. Decreased expression of 2A isoforms of brain-derived neurotrophic factor and N-methyl-D-aspartate (NMDA) receptors in mice deficient in stress response (Worthington et al., 2018). NMDA receptors affect the release and expression of corticotropin-releasing hormone (CRH) in the hypothalamus and induce changes in HPA axis function. At the same time, the stress-HPA axis also affects the composition of the gut microbiota. HPA axis and intestinal microorganisms complement can influence each other, and jointly participate in the pathophysiological process after IS. During dysregulation of the gut microbiota after IS, substances such as cytokines (e.g., IL-1β, IL-6, and TNF-α), are over released and enter the brain via BBB with enhanced permeability and activate the HPA axis. Cortisol, a substance that plays a central role in the stress response, controls its further production by regulating corticotropin in the hypothalamus (Berg and Kaunitz, 2016; Gribble and Reimann, 2016). Therefore, the HPA axis, a key regulator of stress response, can also regulate the gut-brain axis. In the hypothalamus, IL-1 and IL-6 can also induce cortisol release through activation of the HPA axis (El Aidy et al., 2014).

## 4. Therapeutic strategies targeting gut-brain axis of IS

### 4.1. Targeting the crosstalk between the CNS and the peripheral immune system

The crosstalk between the CNS and peripheral immune system in the gut-brain axis provides opportunities for development of new treatment strategies to repair post-IS nerve damage and related research on the treatment of brain-peripheral immune interaction and promoting repair is mainly divided into two aspects: one involves downregulation of pro-inflammatory and downstream pro-apoptotic cascades evoked by this interaction, thereby reducing the infiltration of inflammatory cells and protecting nascent, uninjured brain tissue. The other mechanism involves upregulation of anti-inflammatory factors.

In clinical trials, anti-inflammatory agents have been shown to be beneficial for neurological recovery following IS; They can alleviate neuroinflammation by influencing the peripheral immune system to counter the neurotoxic inflammatory response of immune cells in the brain. Fingolimod is currently used to treat multiple sclerosis, and its primary mechanism is the inhibition of inflammatory T lymphocyte infiltration. Fu et al. (2014) demonstrated that oral fingolimod is safe and possibly beneficial if administered to patients with acute and anterior circulation occlusive stroke within 72 h. Fingolimod is able to limit post-IS secondary tissue damage, reduce microvascular permeability, alleviate neurological deficits, and promote recovery from baseline to 7 days. Another multicenter trial assessed whether combining the immunomodulators fingolimod and alteplase is safe and effective in reducing reperfusion injury in acute IS patients treated within 4.5 h of symptom onset (Zhu et al., 2015). They found that patients who received fingolimod in combination with alteplase exhibited lower circulating lymphocyte counts and smaller lesion volumes than patients who received alteplase alone. They also demonstrated that fingolimod and alteplase combination therapy with alteplase was well tolerated, attenuated reperfusion injury, and improved clinical outcomes in patients with acute IS. These findings should be verified in future clinical trials. In a rat MCAO model, fingolimod improved memory performance after MCAO by LTP induction via postsynaptic mechanisms (Nazari et al., 2016). Fingolimod can also target brain inflammation by skewing microglia toward M2 polarization after chronic cerebral hypoperfusion (Qin et al., 2017; Table 1).

**TABLE 1:** Drugs that targeting gut-brain axis of ischemic stroke (IS).

Strategy	Drug	Results	References
Targeting the crosstalk between the CNS and the peripheral immune system	Fingolimod	Oral fingolimod is safe within 72 h, and limits secondary tissue damage after IS, reduces microvascular permeability, alleviates neurological deficits, and promotes recovery from baseline to 7 days.	Fu et al., 2014
		Whether the combination of the immunomodulators fingolimod and alteplase is safe and effective in reducing reperfusion injury in acute ischemic stroke patients treated within the first 4.5 h of symptom onset.	Zhu et al., 2015
		Fingolimod improved the memory performance after MCAO by LTP induction *via* post-synaptic mechanisms.	Nazari et al., 2016
		Target brain inflammation by skewing microglia toward M2 polarization after chronic cerebral hypoperfusion.	Qin et al., 2017
	Etanercept	Peripheral administration of etanercept significantly improved motor function, and provide significant benefits for the chronic post-stroke management of pain in chronic stroke patients.	Ralph et al., 2020
	Minocycline	The bacteriostatic antibiotic minocycline can activate M2 microglia/macrophage surrounding and within the peri-infarct areas, significantly decrease TNF-α and IL-1β levels, and increase TGF-β, IL-10 levels.	Yang et al., 2015
Activation of AhR	Laquinimod	Reduces the inflammatory response and exerts neuroprotection after IS by inhibiting MPP-induced NLRP3 activation, reducing myelin loss and increasing protective natriuretic factors.	Colombo et al., 2020; Zhang et al., 2020a
	*Lactobacillus reuteri*	Function as an AhR ligand-fermenting probiotic.	Cervantes-Barragan et al., 2017

Another drug, etanercept, is a selective TNF inhibitor and is a promising strategy for targeting neurotoxic immune responses. In a randomized double-blind placebo-controlled clinical trial, peripheral administration of etanercept significantly improved motor function and provided significant benefits for chronic post-stroke pain management in chronic stroke patients (Ralph et al., 2020; Table 1). The bacteriostatic antibiotic minocycline can activate M2 microglia/macrophages surrounding and within the peri-infarct areas, significantly decrease TNF-α and IL-1β levels, and increase TGF-β and IL-10 levels (Yang et al., 2015). If applied in combination with rt-PA treatment, minocycline can reduce the inflammatory response triggered by rt-PA (Switzer et al., 2011).

### 4.2. Activation of AhR

Aryl hydrocarbon receptor, a recognized bioreceptor that maintains intestinal epithelial and immune cell homeostasis, can modulate responses to environmental stimuli. Historically recognized for its role in toxicology, it has been increasingly recognized in recent decades as an important disease modulator, particularly in modulating immune and inflammatory responses. AhR can affect immune and inflammatory responses by targeting gene expression and altering immune differentiation (Neavin et al., 2018; Shinde and McGaha, 2018; Ojo and Tischkau, 2021; Stockinger et al., 2021). Therefore, AhR may serve as a potential therapeutic target in IS. The NLRP3 inflammasome plays an essential role in neuroinflammation during stroke (Alishahi et al., 2019; Sun et al., 2019; Zhu et al., 2021). Laquinimod, an AhR agonist, reduces the inflammatory response and has a neuroprotective effect after IS by inhibiting MPP-induced NLRP3 activation, reducing myelin loss, and increasing protective natriuretic factors (Colombo et al., 2020; Zhang et al., 2020a). *Lactobacillus reuteri*, an AhR ligand-fermenting probiotic, may also be an effective anti-inflammatory agent (Cervantes-Barragan et al., 2017; Table 1). Therefore, AhR receptors can serve as a node that combines signals from the microbiota environment in the gut lumen with physiological signals from the gut neural circuits to maintain the balance of the gut internal environment, and provide new therapeutic ideas for the treatment of IS.

### 4.3. Fecal microbiota transplantation and probiotic intervention

Fecal microbiota transplantation (FMT) is the process of transplanting fecal bacteria from healthy individuals into recipients to relieve clinical symptoms. The efficacy of FMT has been demonstrated in clinical trials with effects ranging from diarrhea to pseudomembranous colitis for the treatment of infections caused by *Clostridium difficile* (Rohlke and Stollman, 2012; Zuo et al., 2018). Additionally, several researchers reported that FMT intervention suppressed neuroinflammation and reduced TLR4 signaling in mouse models of Parkinson’s disease (Sun et al., 2018; Zhao et al., 2021). Kang et al. (2017) described alterations in the intestinal ecosystem that reduced autism symptoms in children with autism spectrum disorders through FMT. These cases suggest that FMT interventions offer new hope for the treatment of various diseases. In recent years, research on the use of FMT interventions to treat post-IS brain injury has also been gradually developed. The gut microbiota and SCFAs metabolism have been shown to have important protective effects against neurodegenerative diseases (Vendrik et al., 2020). Cerebral IS triggers dysfunctional gut microbiota and increases gut permeability. Chen et al. (2019c) showed that oral non-absorbable antibiotics can alter gut microbiota, thereby reducing nerve damage and cerebral infarct volume, relieving cerebral edema, and lowering blood lipid levels. The study also showed that FMT intervention significantly altered the composition of the gut microbiota in IS, which significantly reduced pathogenic bacteria and increased beneficial bacteria. Compared with other SCFAs, butyrate has the highest negative correlation with IS. However, the mechanism of butyrate’s effect on IS is still not fully understood, and it is speculated that it may be through remodeling intestinal microbiota, enriching beneficial microbiota, repairing the leaky intestine and reducing the degree of nerve damage. In addition, butyrate can also relieve brain edema, lower blood lipid levels, and reduce the risk of thrombosis (Chen et al., 2019c). Interference of intestinal microbiota by transplantation of SCFA-rich fecal microbiota and butyric acid supplementation can be considered as an effective treatment for IS (Holmes et al., 2020; Lee et al., 2020; Vendrik et al., 2020; Yuan et al., 2021). Type 2 diabetes (T2D), one of the complications of IS, may aggravate brain injury after IS (Luitse et al., 2012). In a study by Xie et al. (2022) they demonstrated that FMT attenuated cerebral ischemic injury in obese IS rats and the beneficial effects of FMT may be mediated by the attenuation of oxidative stress and apoptosis in the brain. A study by Wang et al. (2021) verified that fecal butyrate-producing bacteria and butyrate levels were significantly lower in Chinese AIS patients with combined T2D than in AIS patients without T2D, and through animal experiments, butyrate can alter the gut microbiota and ameliorate brain injury after acute IS. Furthermore, by transplanting fecal samples from SB-intervened T2D mice into an antibiotic-treated IS mouse model, IS mice receiving gut microbiota from butyrate-treated mice had smaller cerebral infarct volumes and reduced serum levels of LPS, LBP, and pro-inflammatory cytokines and improved BBB permeability compared to controls. These findings suggest that SB supplementation, or FMT, is a potential therapeutic strategy for IS patients combining T2D.

Malnutrition is a common complication after IS, which is strongly associated with poor outcomes and has led to a number of studies targeting stroke-related probiotic interventions (Wu and Chiou, 2021). A systematic review discussed that probiotic supplementation, both for single and multiple bacterial species, significantly reduced body weight, BMI, TNF-α, total cholesterol, and LDL cholesterol levels compared to controls (Savigamin et al., 2022). A systematic Review and Meta-analysis consumption of fermented milk was associated with a 4% reduction in risk of stroke and cardiovascular death, while an analysis of 52 RCTs illustrated that probiotic-added dairy products were associated with better outcomes compared to taking only probiotic capsules or powders (Companys et al., 2020). Several research demonstrated that two types of food supplements, probiotics and prebiotics, have the potential to mitigate the risk of cardiovascular disease by improving total and LDL cholesterol, high-sensitivity C-reactive protein (hs-CRP), and other certain cytokines (such as TNF-α and IL-1β) involved in the inflammatory response (Gomes et al., 2017; Raygan et al., 2018; Hassan et al., 2020). One RCT showed that probiotics had a significant reduction in the risk of cardiovascular disease at supplemental doses of no less than 10^10^ colony forming unit and with an intervention duration of no less than 8 weeks (Liu et al., 2021). Therefore, early nutritional support and long-term probiotic interventions are particularly important for stroke patients, and probiotic supplementation may be effective in improving the clinical prognosis of IS patients, especially nasal feeding IS patients, while reducing the incidence of infectious events and various related complications (Liu et al., 2021). A study investigated the effects of *Clostridium butyricum*, a type of probiotics, on cerebral I/R injury in diabetic mice subjected to 30 min of bilateral common carotid artery occlusion. The result suggests that *C. butyricum* can reduce cognitive impairment, reverse the cerebral ischemia/reperfusion (I/R) induced decreases in p-Akt expression and increases in caspase-3 expression leading to inhibition of neuronal apoptosis (Sun et al., 2016b). Several studies have shown that probiotic supplementation reduces cerebral infarct size, decreases TNF-α levels, and prevents spatial learning and memory deficits in a mouse model of IS (Akhoundzadeh et al., 2018; Rahmati et al., 2019). However, the efficacy of fecal microbiota transplantation and probiotics or dietary fiber supplementation in patients with IS needs to be tested further with clinical trials.

## 5. Conclusion

**FIGURE 1:**
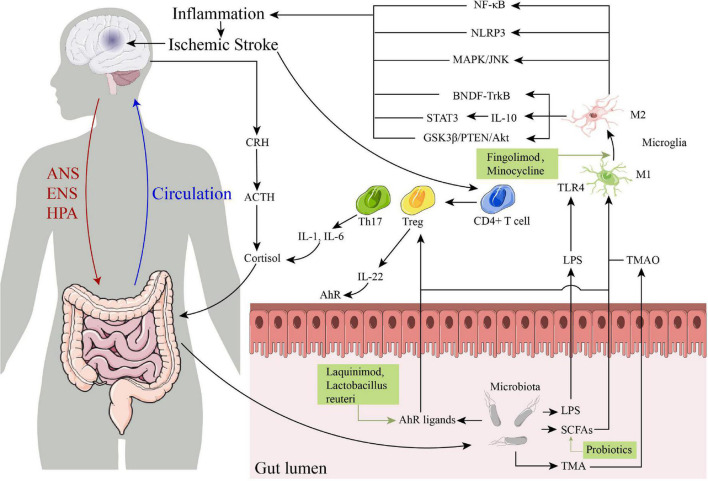
The specific immune-related pathways in the post-ischemic stroke (IS) gut-brain axis and some drugs that targeting gut-brain axis of IS. ANS, autonomic nervous system; ENS, enteric nervous system; HPA, hypothalamic-pituitary-adrenal; CRH, corticotropin-releasing hormone; ACTH, corticotropin-releasing hormone; IL, interleukin; TLR4, toll-like receptor 4; LPS, lipopolysaccharide; SCFAs, short-chain fatty acids; TMA, trimethylamine; TMAO, trimethylamine N-oxide; NF-κB, nuclear factor kappa B; NLRP3, NOD-like receptor thermal protein domain associated protein 3; AhR, aryl hydrocarbon receptor.

The drugs that targeting gut-brain axis of IS are schematically summarized in Table 1, and the specific immune-related pathways in the post-IS gut-brain axis are summarized in Figure 1. The gut-brain axis dysfunction after stroke is a promising area of research for identifying novel mechanisms and strategies for both prevention and treatment of stroke. After IS, damage to the gut-brain axis leads to DAMPs, cytokine release, changes in the BBB, altered or dysregulated microbiota composition, and leaky gut, leading to the migration of inflammatory and immune cells from the gut to the brain through the following pathways and interaction with innate immune cells in the CNS. In this process, γδ Tregs, IL-17, IL-6, and IL-1β are decreased, while Tregs and IL-10 are increased, which reduces pro-inflammatory effects and promotes anti-inflammatory processes after IS. Several pathways are involved in the gut-brain axis after IS, including the LPS/TLR4, SCFA receptor, and AhR signaling pathways and the TMAO/NLRP3 and HPA axis pathways. The resulting treatment strategies mainly include targeting the crosstalk between the CNS and the peripheral immune system (using drugs such as fingolimod, etanercept, minocycline), activation of AhR (using laquinimod, *Lactobacillus reuteri*), fecal microbiota transplantation and probiotic intervention, which may provide new therapeutic strategies for brain injury after IS and reduce the poor prognosis of IS patients.

The current researches on the gut microbiota of the gut-brain axis and the related mechanisms are still in the initial stage. Existing studies suggest that the gut microbiota has greater genetic diversity than the host and may be an important factor in determining the development and prognosis of IS disease and the effectiveness of drug therapy. At the same time, even for microorganisms of the same genus, their different species and strains may have different effects on different processes of the same disease, therefore, a multi-omics approach can be used to detect the relevant potential pathogenic agents after stroke through human disease or animal models microorganisms, metabolites and their characteristics can be identified by identifying the cellular pathways and cellular functions targeted by their targets, integrating the information and revealing the deep-seated molecular mechanisms to develop individualized medical treatment for future stroke diseases. This area also needs the establishment of clinical cohort studies for the collection of data to build health and disease databases and the development of clinical treatments and standard operating protocols. Besides, some unresolved issues remain. For example, changes to the BBB and post-IS gut-induced inflammatory and immune responses as well as gut leakage and dysbiosis after stroke, are topics that would benefit from further study. Moreover, the exact molecular mechanisms underlying changes in the gut-brain axis require further investigation. Further clinical studies are needed to shed light on how gut dysbiosis affects peripheral immune responses and stroke outcomes, and whether dietary supplements have the potential to address any of these issues.

## Author contributions

S-YZ searched the literature and drafted the manuscript. Z-NG, YY, YQ, and HJ critically revised the manuscript. All authors have made contributions to the work and approved it for publication.
